# Regulation of sperm motility in Eastern oyster (*Crassostrea virginica*) spawning naturally in seawater with low salinity

**DOI:** 10.1371/journal.pone.0243569

**Published:** 2021-03-18

**Authors:** Zoe G. Nichols, Scott Rikard, Sayyed Mohammad Hadi Alavi, William C. Walton, Ian A. E. Butts

**Affiliations:** 1 School of Fisheries, Aquaculture and Aquatic Sciences, Auburn University, Auburn, Alabama, United States of America; 2 Auburn University Shellfish Lab, Dauphin Island, Alabama, United States of America; 3 School of Biology, College of Science, University of Tehran, Tehran, Iran; Cinvestav-IPN, MEXICO

## Abstract

Oyster aquaculture is expanding worldwide, where many farms rely on seed produced by artificial spawning. As sperm motility and velocity are key determinants for fertilization success, understanding the regulation of sperm motility and identifying optimal environmental conditions can increase fertility and seed production. In the present study, we investigated the physiological mechanisms regulating sperm motility in Eastern oyster, *Crassostrea virginica*. Sperm motility was activated in ambient seawater with salinity 4–32 PSU with highest motility and velocity observed at 12–24 PSU. In artificial seawater (ASW) with salinity of 20 PSU, sperm motility was activated at pH 6.5–10.5 with the highest motility and velocity recorded at pH 7.5–10.0. Sperm motility was inhibited or totally suppressed in Na^+^, K^+^, Ca^2+^, and Mg^2+^-free ASW at 20 PSU. Applications of K^+^ (500 μM glybenclamide and 10–50 mM 4-aminopyridine), Ca^2+^ (1–50 μM mibefradil and 10–200 μM verapamil), or Na^+^ (0.2–2.0 mM amiloride) channel blockers into ASW at 20 PSU inhibited or suppressed sperm motility and velocity. Chelating extracellular Ca^2+^ ions by 3.0 and 3.5 mM EGTA resulted in a significant reduction and full suppression of sperm motility by 4 to 6 min post-activation. These results suggest that extracellular K^+^, Ca^2+^, and Na^+^ ions are involved in regulation of ionic-dependent sperm motility in Eastern oyster. A comparison with other bivalve species typically spawning at higher salinities or in full-strength seawater shows that ionic regulation of sperm motility is physiologically conserved in bivalves. Elucidating sperm regulation in *C*. *virginica* has implications to develop artificial reproduction, sperm short-term storage, or cryopreservation protocols, and to better predict how changes in the ocean will impact oyster spawning dynamics.

## Introduction

Similar to vertebrates [1–4], sperm of most invertebrates including bivalves are non-motile in the testis and reproductive tract [5]. Bivalves usually possess an external fertilization strategy, where sperm motility is activated after expulsion into the aquatic environment. Compared to other aquatic organisms including echinoderms, tunicates [6–8], fishes [4,9,10], and amphibians [2,11], regulation of sperm motility is largely unknown in bivalves [5].

Once sperm motility in aquatic animals is activated by environmental osmolality or ions [4–13], sperm subsequently have a limited period of motility to reach an oocyte [4,14,15]. This is due to rapid depletion of energy required for the activity of the sperm motility apparatus called the axoneme [4,16]. In addition to sperm production [17–19], sperm motility kinematics including the percentage of motile sperm and sperm velocity are key determinants for successful fertilization [14,17,20]. Sperm quality can vary among wild and cultivated specimens and even displays male-to-male variability [21–24]. Sperm motility kinematics can be assessed using computer-assisted sperm analysis (CASA) software that tracks sperm head trajectories [25].

To understand regulation of sperm motility, it is essential to investigate the physiological status that maintains sperm in the quiescent state in the reproductive organ and activates sperm motility before and after discharge into the aquatic environment, respectively. Ions including proton (H^+^), calcium (Ca^2+^), potassium (K^+^), magnesium (Mg^2+^), and sodium (Na^+^) play critical roles in regulation of sperm motility [1,2,4,6,9,12,13]. In these contexts, osmolality-, pH-, and ionic-regulation of sperm motility can be understood by decreasing or increasing these parameters compared to those physiologically found in testicular fluid. The effects can also be analyzed by completely removing the ions from the sperm activating medium, or by applying a proper channel blocker which inhibits ionic fluxes across the sperm membrane. So far, it has been shown that environmental osmolality, pH, and ions are involved in sperm motility regulation in many aquatic organisms, including echinoderms, tunicates, bivalves, and fishes [4–10]. In general, low pH, high K^+^ concentrations, or osmolality of the testicular (seminal) fluid controls sperm motility in the reproductive organ of aquatic animals. In aquatic vertebrates (amphibian and fish), sperm motility is mostly regulated by hypoosmolality- and hyperosmolality-induced ionic fluxes in freshwater and marine species, respectively [2,4,6,9]. In aquatic invertebrates including bivalves, sperm motility is osmotic-independent as there is no difference in osmolality between the testicular fluid and the aquatic environment [5,7,8]. Therefore, regulation of sperm motility shows high diversity among aquatic organisms. It is also worth noting that some endogenous inhibitory (e.g. peptides) or stimulatory (e.g. egg-derived peptides, serotonin or steroids) factors for sperm motility have been also identified [26–29].

The mollusks have recently become a major taxonomic group for commercial fisheries and aquaculture, where global production has increased from 3.6 to 17.1 million tons between 1990 and 2016 [30]. Artificial seed production is vital to expand aquaculture. Among 15,000 bivalve species from the phylum “Mollusca” possessing about 100,000 species [31], sperm motility has only been studied in a few ecologically and economically important species that typically spawn at higher salinities or in full-strength seawater, including the Atlantic surf clam (*Spisula solidissima*) [32], Pacific oyster (*Crassostrea gigas*) [28,33–36], black-lip pearl oyster (*Pinctada margaritifera*) [37], Japanese pearl oyster (*Pinctada fucata martensii*) [38], European flat oyster (*Ostrea edulis*) [39], Manila clam (*Ruditapes philippinarum*) [28], great scallop (*Pecten maximus*) [40], and Japanese scallop (*Patinopecten yessoensis*) [28]. To the best of our knowledge, there is limited information available regarding sperm motility for the Eastern oyster, *Crassostrea virginica* [41]; a euryhaline species whose optimal salinity range for growth and reproduction is generally considered to be ~10 to 30 PSU [42–44].

In the Pacific oyster [45], it has been reported that low salinities reduced fertilization and larval development that might be related to inhibition of sperm motility [33,36]. In the Eastern oyster [46], it has been reported that low environmental pH inhibits spawning. Further studies have shown that sperm motility was inhibited and initiated in bivalves at acidic and alkaline pH, respectively [28,33,36–38,40]. These reports suggest that inhibition of spawning at low salinity or pH might be due, at least in part, to their effects on sperm motility. Therefore, acidic pH of testicular fluid in bivalves may contribute to maintain sperm in the quiescent state in the reproductive organ [28,36,37]. Alavi et al. [28] reported that K^+^ concentration is high in the testicular fluid of Pacific oyster and Japanese scallop, and a K^+^ efflux via a voltage-dependent K^+^ channel was suggested after discharge of sperm into seawater. Contribution of an ATP-sensitive K^+^ channel to trigger sperm motility activation in bivalves is unclear but has been reported in fish sperm [47,48]. Similar to that of other aquatic vertebrates or invertebrates [49–54], sperm motility activation in bivalves required Ca^2+^ influx as chelating extracellular Ca^2+^ ([Ca^2+^]_e_) ions or application of Ca^2+^ channel blockers resulted in suppression or inhibition of sperm motility [28]. Activation of sperm motility in bivalves was also inhibited in Na^+^-free seawater, and a Na^+^/Ca^2+^ or a Na^+^/H^+^ exchanger are likely involved in regulation of intracellular pH ([pH]_i_) and intracellular Ca^2+^ ([Ca^2+^]_i_) [28,36,40,55,56]. Among ions, Mg^2+^ may not play a key role in sperm motility signaling since Pacific oyster sperm motility has been initiated in Mg^2+^-free seawater [36]. Taken together, these studies illustrate how sperm motility activation occurs in bivalve species that typically spawn at higher salinities or in full-strength seawater, but still raises the question as to whether it will follow a similar trend for a species that can spawn at lower salinities.

The Eastern oyster is native to benthic habitats along the Atlantic coast of America from Canada to the Gulf of Mexico, the Caribbean, and the coast of South America [57]. It is a keystone species with many ecological services and economic value [58]. Since the mid 1800s, wild oyster populations in the Mid-Atlantic and Gulf Coast of the USA have declined due to a combination of overharvesting, habitat degradation, disease, and poor water quality [57]. Restoration efforts are hindered by increasing land development and fatal diseases, which devastate oyster populations and recruitment [58–60]. Widespread diseases and the availability and quality of seed to farms are bottlenecks for oyster production [61,62]. Historically, the oyster industry relied on collecting wild spat from the environment, but anthropogenic impacts such as the Deepwater Horizon oil spill in 2010 have negatively affected reproductive success [63]. Thus, contemporary research is now directed towards artificial spawning and cryopreservation to supply oyster seed to farms [64].

In the present study, our goal was to investigate regulation of sperm motility in the Eastern oyster, where specific objectives were to study the contributions of salinity, pH, and ions. In this context, we evaluated sperm motility and velocity after activating sperm in ambient seawater and artificial seawater (ASW) with different salinity and pH, respectively. To understand the contribution of ions in regulation of sperm motility, sperm were activated in K^+^, Ca^2+^, Na^+^, and Mg^2+^-free ASW or in ASW with various ion channel blockers. Thereafter, sperm kinematics (motility and velocity) were quantified using a CASA system. Our results provide valuable information to develop methods for artificial reproduction, short- or long-term preservation of sperm, and to understand species specificity of sperm motility regulation in bivalves that might be related to environmental changes.

## Materials and methods

### Ethics

All investigations using male Eastern oyster were conducted in accordance with the Auburn University Animal Care and Use Program (IACUC).

### Chemicals

Buffers including MES, HEPES, and Tris were obtained from VWR Life Science as well as ethylenediaminetetraacetic acid (EDTA). Nifedipine and choline chloride were from Beantown Chemical. Pluronic F-127 and mibefradil dihydrochloride hydrate were from Sigma Aldrich. Verapamil hydrochloride, glybenclamide, 4-Aminopyridine (4-AP), amiloride hydrochloride dehydrate, NaOH, HCl, ethylene glycol-bis(β-aminoethyl ether)-N,N,N′,N′-tetraacetic acid (EGTA), and DMSO were purchased from AdipoGen, InvivoGen, Acros Organics, ENZO, Fisher Scientific, EMD, Alfa Aesar, and Macron Fine Chemicals, respectively. KCl, CaCl_2_ × 2H_2_O, MgCl_2_ × 6H_2_O, and MgSO_4_ × 7H_2_O were purchased from VWR Analytical, Ward’s Science, EMD Millipore Corp, and Ward’s Science, respectively.

### Oysters and sperm collection

Oysters were collected in batches from floating cages and longline systems at the Auburn University Shellfish Laboratory (AUSL) research oyster farm site in Grand Bay, AL (30°22’34.8” N 88°18’52.8” W), and transported to the AUSL in Dauphin Island, AL (30°14’51.9” N, 88°4’46.9” W) or the Aquatic Reproductive Physiology Laboratory (ARPL) at Auburn University, Auburn, AL. At AUSL, the oysters were held in a flow-through system at 19°C and 10 PSU. The incoming seawater flowed through a 50-micron bag filter and was chilled with a 1/3 HP Trimline Delta Star Water Chiller. At the ARPL, oysters were acclimated and held in a recirculation aquaculture system, equipped with two 794 L polyethylene round open top tanks (122 cm diameter × 81 cm height) (Dura-Cast Products, Inc., Lake Wales, FL). Each day, the oysters were fed ~40 mL of Shellfish Diet 1800 (Reed Mariculture Inc., Campbell, CA). Seawater was made by mixing Crystal Sea Marinemix (Marine Enterprises International, LLC, Baltimore, MD) into tap water (originating from deep well aquifers and springs via the City of Auburn, AL) filtered through a reverse osmosis system (Barracuda Glacial 100 GPD RO/DI, AquaFX, Winter Park, Florida) to reach a salinity of 10 PSU that was identical to environmental salinity at the collection site. Water temperature was also maintained at 19°C.

Oysters were opened with a shucking knife, cup-side down, at the hinge, and sex was determined by observation of the gonad samples under a microscope. Each male was then thoroughly rinsed with distilled water to flush away any excess seawater. The mantle tissue around the gonad was dried with paper towels before semen collection by pipette. Collected semen was stored in a 1 mL Eppendorf tube and placed into a chilling block (EchoTherm™ IC50, Torrey Pines Scientific, Inc., Carlsbad, CA, USA) set to 19°C. An aliquot of each sample was examined to ensure the sperm was not moving prior to the start of each experiment. If the sperm was motile (an indication that semen was contaminated with seawater) the sample was discarded. Morphometrics of oysters including length, width, and height (± 0.01 mm) were recorded as well as sperm density using a Neubauer hemocytometer (S1 Table).

### Testicular fluid osmolality and pH

Osmolality of testicular fluid (n = 5 males) was determined in triplicate for each sample using a Vapor Pressure Osmometer (Model 5600 Wescor, Inc, Logan, Utah, USA). The pH of testicular fluid (n = 5 males) was measured using a benchtop pH meter (Orion Star™ A111 Benchtop pH Meter, Thermo Fischer Scientific, Madison, WI, USA) and an electrode (Orion™ PerpHecT™ ROSS™ Combination pH Micro Electrode, Thermo Fischer Scientific, Madison, WI, USA). Osmolality and pH were also measured for all activation media.

### Assessment of sperm kinematics

In all experiments, sperm activating solutions (500–1000 μL) were pipetted into 1.5 mL Eppendorf tubes placed into a chilling block set to 19°C for the duration of the experiment. To activate sperm motility, 0.2–1.0 μL of semen was then diluted with sperm activating solutions in the Eppendorf tube and inverted several times to mix thoroughly. At different times post-activation, 5 μL of suspension was pipetted into a 20 μm deep 2X-CEL chamber (Hamilton Thorne Biosciences, Beverly, MA, USA), placed under a light microscope (AX10 Lab.A1, Carl Zeiss Meditec Inc., CA, USA), and sperm motility was recorded at 20× magnification. Percentage of motile sperm and curvilinear velocity (VCL) were determined. All solutions contained 0.4% Pluronic F-127 to prevent sperm from sticking to the slides [28].

### Effect of salinity on sperm kinematics

Semen of 5 males were used to study the effect of salinity on sperm motility. Ambient seawater from the AUSL was boiled to make a high salinity stock solution (40 PSU). This stock solution was then cooled and diluted with ultrapure water to make salinities ranging from 4 to 32 PSU. A refractometer and a Vapor Pressure Osmometer were used to determine salinity and osmolality, respectively (S2 Table). Seawater with different salinities were buffered with 20 mM Tris, pH 8.0 ± 0.2. Salinities were checked prior to the start of the experiments.

### Effect of pH on sperm kinematics

Semen of 4 males were used to study the effect of pH on sperm motility and velocity. Artificial seawater (ASW) was made with 516 mM NaCl, 10.4 mM KCl, 11 mM CaCl_2_, 34 mM MgCl_2_, and 22 mM MgSO_4_ [36]. Salinity of this ASW was 40 PSU and sperm were immotile in it. Salinity of ASW was adjusted to 20 PSU using ultrapure water (Millipore Direct Q 3 UV) in which sperm motility was fully initiated. The ASW at 20 PSU was buffered with 20 mM MES, HEPES or Tris and pH was adjusted between 5.0 and 11.0 using NaOH or HCl. MES, HEPES, and Tris were used to buffer pH of 5.0–6.0, 6.5–8.0, and 8.5–11.0, respectively.

### Effect of ions on sperm kinematics

Semen of 4–5 males were used to study the effect of ions on sperm motility and velocity. The ASW without KCl or CaCl_2_ × 2H_2_O was made as a K^+^-free ASW or Ca^2+^-free ASW, respectively. The ASW without MgCl_2_ × 2H_2_O and MgSO_4_ × 7H_2_O was made as a Mg^2+^-free ASW. In this solution, choline chloride (56 mM) was used as a substitute for MgCl_2_ and MgSO_4_. ASW containing 516 mM choline chloride as a substitute for NaCl was made as Na^+^-free ASW. All of the above activating solutions were buffered with 20 mM Tris to a pH of 8.0 ± 0.2 [28,36]. Initial salinity of ASW was 40 PSU then diluted by half with ultrapure water to reach a salinity of 20 PSU. The controls for these experiments were ASW with and without DMSO or ion-free ASW with and without DMSO.

To examine the effects of K^+^ on sperm motility and velocity, sperm motility was activated in K^+^-free ASW. To elucidate K^+^ ion fluxes, K^+^ channel blockers (glybenclamide and 4-AP) were used. Glybenclamide and 4-AP were dissolved in DMSO and in ultrapure water, respectively. Concentrations of stock solutions for glybenclamide was 5 and 50 mM and for 4-AP was 100, 1000, and 1500 mM. The DMSO contribution to the control solutions was 1% to represent the DMSO level in the highest channel blocker treatment. All channel blockers were added to ASW.

To study the effects of Ca^2+^ on sperm motility and velocity, sperm motility was activated in Ca^2+^-free ASW with or without EGTA that chelate out any trace of Ca^2+^ ions. Various concentrations of EGTA (0.5–3.5 mM) were examined from a 100 mM stock solution of EGTA dissolved in distilled water and buffered with 20 mM Tris, pH 8.0 ± 0.2. To study Ca^2+^ flux in sperm motility, sperm was activated in 20 PSU ASW containing various Ca^2+^ channel blockers (mibefradil, verapamil, and nifedipine). Stock solutions of 10 mM mibefradil, 50 mM verapamil, and 50 mM nifedipine were made. Mibefradil was dissolved in ultrapure water, while verapamil and nifedipine were dissolved in DMSO. The DMSO contribution to the control solutions was 0.04%.

To study the effects of Na^+^ on sperm motility and velocity, sperm motility was activated in Na^+^-free ASW. To elucidate Na^+^ ion fluxes, a Na^+^ channel blocker (amiloride) was used. Amiloride was dissolved in DMSO with stock concentrations of 20 and 200 mM. The DMSO contribution to the control solutions was 1% to represent the DMSO level in the highest channel blocker treatment. Amiloride was added to ASW and Na^+^-free ASW solutions.

To study the effects of Mg^2+^ on sperm motility and velocity, sperm motility was activated in Mg^2+^-free ASW with or without 1 mM of EDTA.

Effective concentrations of ion channel blockers were determined according to previous studies that have used similar chemicals [28,47,49–51,54–56] due to no molecular identity of ion channels in sperm of bivalves.

### Statistical analyses

Data were analyzed using SAS statistical analysis software (v. 9.1; SAS Institute Inc., Cary, NC, USA). Residuals were tested for normality (Shapiro-Wilk test) and homogeneity of variance (plot of residuals *vs*. predicted values). To meet assumptions of normality and homoscedasticity, when necessary, sperm velocity data were log_10_ transformed and sperm motility data were arcsine square-root transformed. The Kenward–Roger procedure was used to approximate denominator degrees of freedom for all F-tests [65]. To examine the effects of pH, salinity, and ions on sperm motility and velocity, data were analyzed using a series of repeated measures factorial ANOVA models. Each model contained the activation media (pH, salinity, or ions), time post-activation, and their interaction (activation media × time post-activation). If an interaction was significant, the models were decomposed into individual one-way ANOVA models at each time post-activation. In the case of a non-significant interaction, the main effects of activation media (pH, salinity, or ions) and time post-activation were interpreted. Treatment means were contrasted using the Tukey’s test. Results were shown as mean ± standard error. Quadratic regressions were used to relate sperm kinematics to salinity and pH of the activation media. Environmental conditions yielding the highest motility and velocity were determined using the first derivative of the quadratic equation set to 0. Alpha was set at 0.05.

## Results

### Testicular fluid osmolality and pH

The testicular fluid osmolality and pH ranged from 476.33 to 682.67 mOsmol/kg (average 569.60 ± 34.60, n = 5) and from 5.81 to 5.99 (average 5.80 ± 0.04, n = 5), respectively.

### Effect of salinity on sperm kinematics

**Fig 1:**
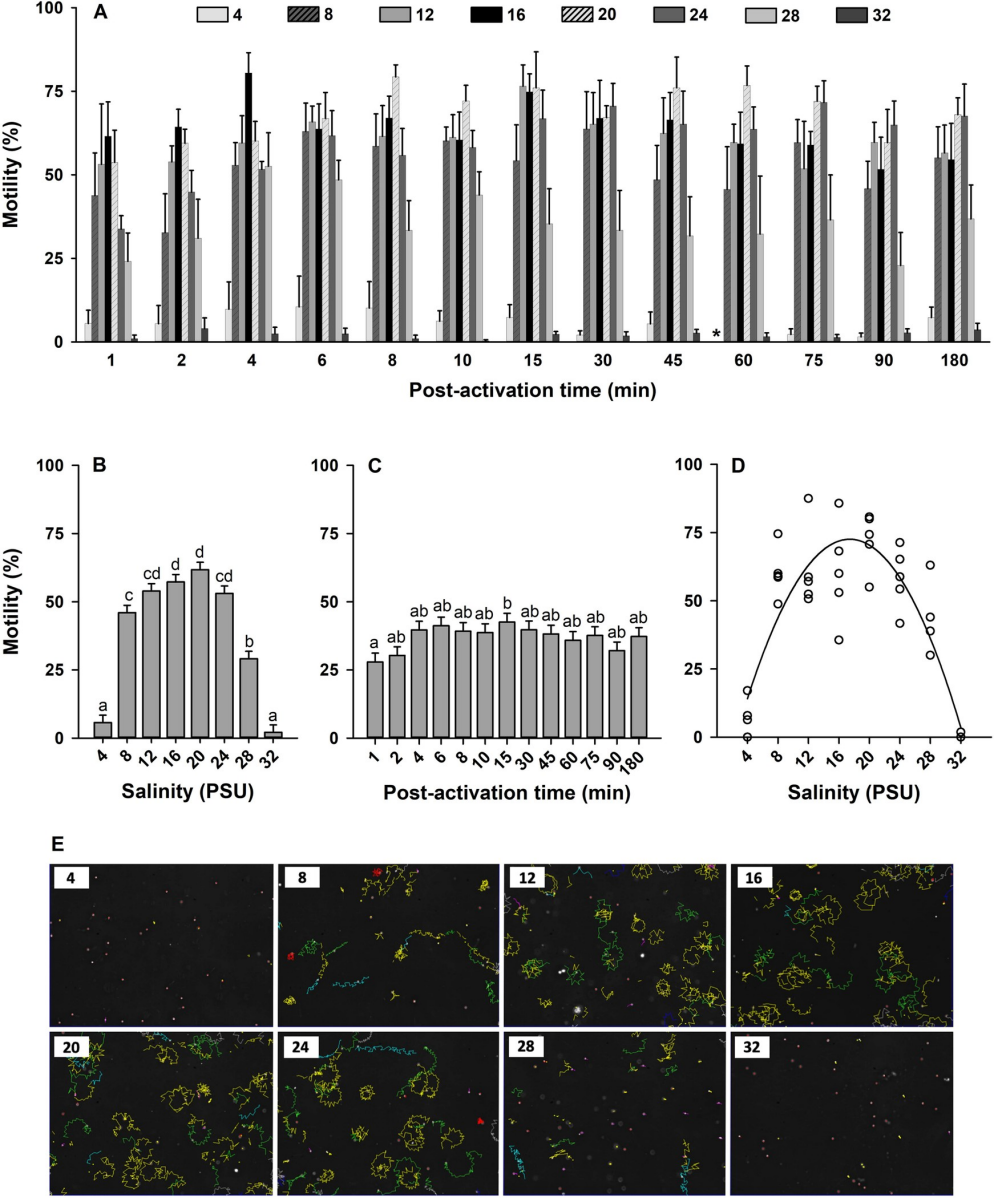
Effect of salinity on sperm motility (%, A) in Eastern oyster, *Crassostrea virginica*. Sperm motility was activated in high salinity seawater diluted to salinities of 4 to 32 PSU. Average motility at each salinity (B) and time post-activation (C) is displayed. D shows a second-order polynomial regression for the effects of salinity on sperm motility at 15 min post-activation. Sperm head trajectories at each salinity are shown (E). Data were analyzed using a repeated measures ANOVA and shown as mean ± SE (n = 5). Treatments with different superscripts significantly differ (*p* < 0.05). A motility of 0% was indicated by asterisk.

**Fig 2:**
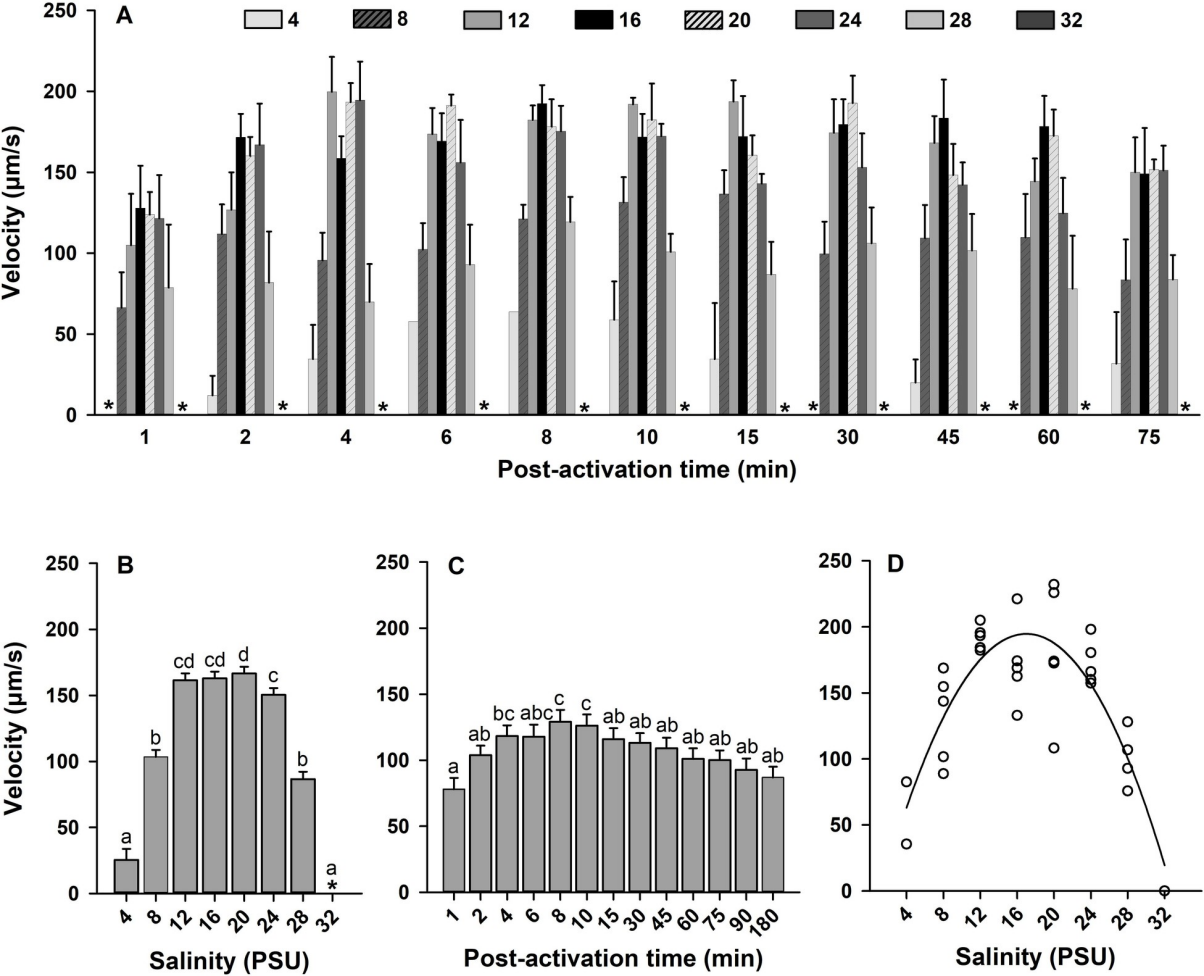
Effect of salinity on sperm velocity (μm/s, A) in Eastern oyster, *Crassostrea virginica*. Sperm motility was activated in high salinity seawater diluted to salinities of 4 to 32 PSU. Average velocity at each salinity (B) and time post-activation (C) is displayed. D shows a second-order polynomial regression for the effects of salinity on sperm velocity at 15 min post-activation. Data were analyzed using a repeated measures ANOVA and shown as mean ± SE (n = 5). Treatments with different superscripts significantly differ (*p* < 0.05). A velocity of 0 μm/s was indicated by asterisk.

To study the effects of salinity on sperm motility, sperm were diluted in seawater with different salinities. Sperm motility was activated across a wide range of salinities between 4 and 32 PSU (Figs 1 and 2). The interaction between salinity × time post-activation was not significant in the model for sperm motility and velocity (*p* > 0.05; Figs 1A and 2A). Therefore, the main effects of salinity and time post-activation were interpreted for both sperm traits at all times collectively.

Salinity of seawater affected sperm motility and velocity with highest values observed in seawater with salinity ranging from 12 to 24 PSU (*p* < 0.0001; Figs 1B and 2B). Sperm motility and velocity were also different at the various times post-activation (*p* < 0.01 and *p* < 0.0001, respectively; Figs 1C and 2C). Relative to 1 min post-activation, significant increases in sperm motility and velocity were observed at 15 min and 4, 8, 10 min, respectively. To determine at which salinity sperm displayed the highest sperm motility and velocity, a series of regressions were generated at each post-activation time for each salinity. The regressions were nonlinear second-order polynomial dome-shaped functions showing highest sperm motility between salinity 16.83 and 17.91 PSU (Fig 1D) and highest sperm velocity between salinity of 16.50 and 18.32 PSU (Fig 2D) from 1 to 180 min post-activation (S3 Table). Sperm motility showed circular trajectories for all salinities indicating that flagellar beating was asymmetric (Fig 1E).

These results show that sperm motility in Eastern oyster was highly inhibited in seawater at 4 PSU and suppressed in full-strength seawater >32 PSU. Further experiments were conducted in ASW with salinity of 20 PSU.

### Effect of pH on sperm kinematics

**Fig 3:**
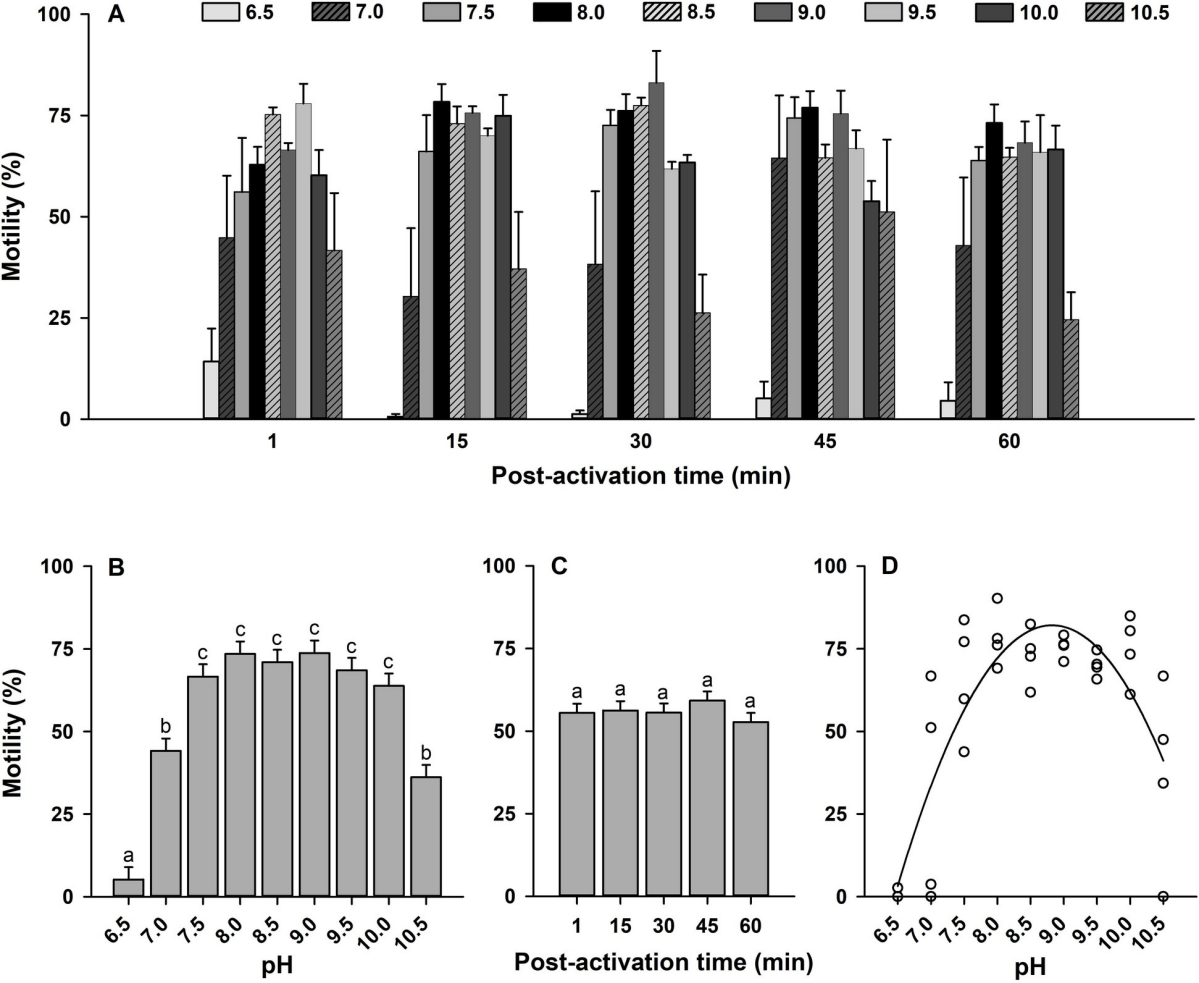
Effect of pH on sperm motility (%, A) in Eastern oyster, *Crassostrea virginica*. Sperm motility was activated in artificial seawater buffered with 20 mM MES, HEPES or Tris, pH 6.5–10.5. Average motility at each pH (B) and time post-activation (C) is displayed. D shows a second-order polynomial regression for the effects of pH on sperm motility at 15 min post-activation. Data were analyzed using a repeated measures ANOVA and shown as mean ± SE (n = 4). Treatments with different superscripts significantly differ (*p* < 0.05). A motility of 0% was indicated by asterisk.

**Fig 4:**
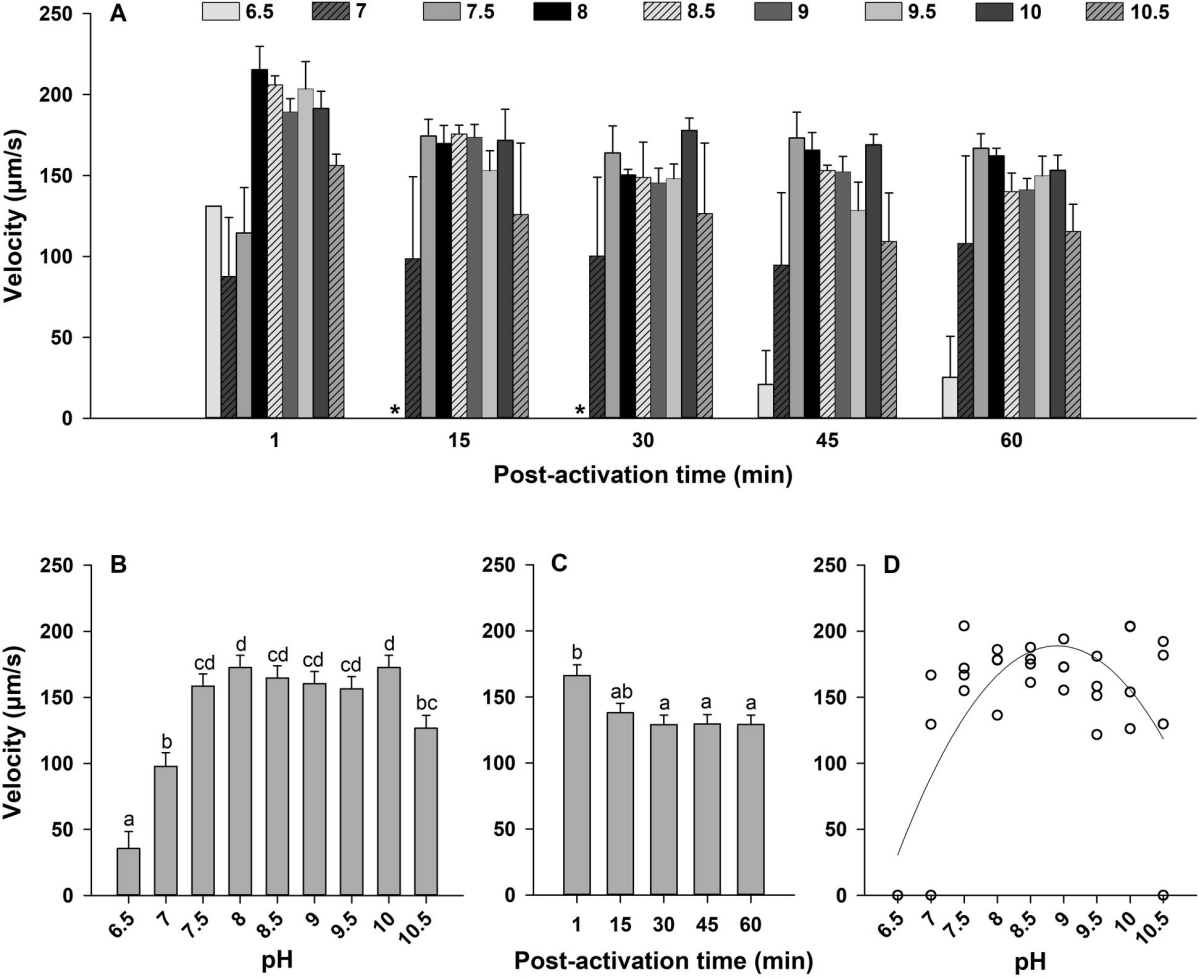
Effect of pH on sperm velocity (μm/s, A) in Eastern oyster, *Crassostrea virginica*. Sperm motility was assessed in artificial seawater buffered with 20 mM MES, HEPES or Tris, pH 6.5–10.5. Average motility at each pH (B) and time post-activation (C) is displayed. D shows a second-order polynomial regression for the effects of pH on sperm velocity at 15 min post-activation. Data were analyzed using a repeated measures ANOVA and shown as mean ± SE (n = 4). Treatments with different superscripts significantly differ (*p* < 0.05). A velocity of 0 μm/s was indicated by asterisk.

The effects of pH on sperm motility were assessed after diluting sperm with ASW at different pH. Sperm motility was activated across a wide range of ASW pH between 6.5 and 10.5 (Figs 3 and 4). In this analysis, interactions between the main factors (pH × time post-activation) showed no significant effects on sperm motility and velocity (*p* > 0.05; Figs 3A and 4A). Therefore, like before, the main effects of pH and time post-activation were interpreted for both sperm traits at all times collectively.

The pH of ASW affected sperm motility and velocity with highest values observed at pH 7.5–10.0 (*p* < 0.0001; Figs 3B and 4B). Sperm motility did not differ with time post-activation (*p* > 0.05; Fig 3C). However, there was an impact for velocity (*p* < 0.01; Fig 4C), in which sperm velocity was highest at 1 min and decreased at 30 min post-activation.

To determine which pH sperm displayed highest motility and velocity, a series of regressions were generated at each time post-activation for each pH. Like the model for salinity, the regressions were nonlinear second-order polynomial dome-shaped functions. Relationships from the model showed that highest sperm motility was between pH 8.66 and 8.83 (Fig 3D) and highest sperm velocity between pH of 8.83 and 9.09 (Fig 4D) from 1 to 60 min post-activation (S3 Table).

These results show that Eastern oyster sperm requires an alkaline condition to exhibit the highest sperm kinematics. Sperm motility was not activated in ASW at pH ≤6.0 and ≥11.0.

### Effect of K^+^ on sperm kinematics

**Fig 5:**
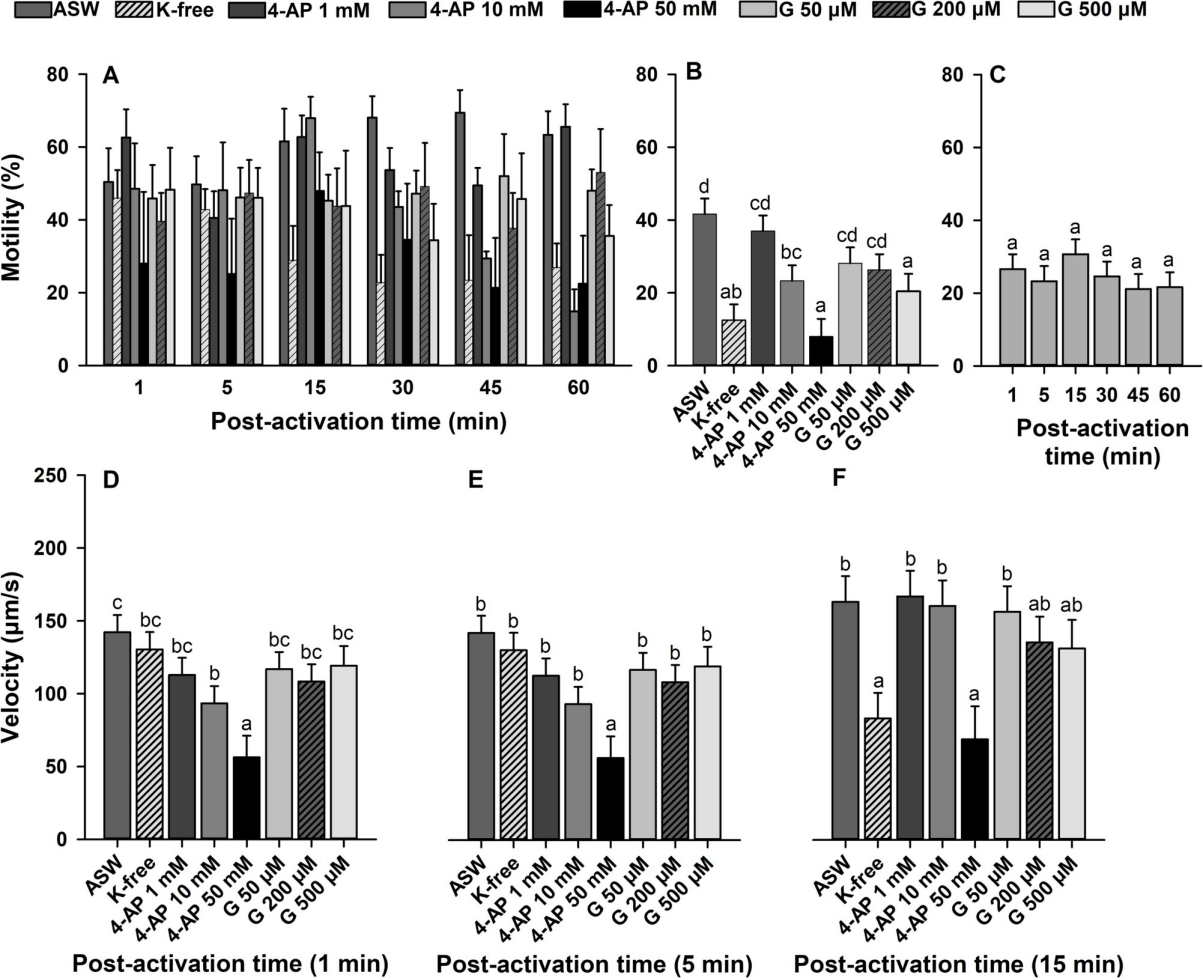
Effect of potassium (K^+^) ions on sperm motility (%, A-C) and velocity (μm/s, D-F) in Eastern oyster, *Crassostrea virginica*. Sperm was activated in K^+^-free artificial seawater (ASW) and ASW containing a voltage-gated (4-aminopyridine, 4-AP) or an ATP-sensitive (glybenclamide, G) K^+^ channel blocker. Average motility in each treatment (B) and time post-activation (C) is displayed. Data were analyzed using a repeated measures ANOVA and shown as mean ± SE (n = 5). Treatments with different superscripts significantly differ (*p* < 0.05).

To understand the contribution of K^+^ ions in sperm motility regulation, sperm were diluted in K^+^-free ASW and in ASW containing K^+^ channel blockers. The interaction between the main factors (K^+^ treatments × time post-activation) was not significant for sperm motility (*p* > 0.05; Fig 5A). Therefore, the effects of K^+^ treatments and time post-activation on sperm motility were interpreted. There were effects of K^+^ treatments on sperm motility (*p* < 0.0001). Motility was decreased in K^+^-free ASW, and ASW containing 4-AP and glybenclamide in a concentration-dependent manner (Fig 5B). Significant decreases in sperm motility were observed at ≥10 mM 4-AP and at 500 μM glybenclamide. However, differences in sperm motility were not significant at various times post-activation (*p* > 0.05; Fig 5C).

For sperm velocity, the interaction between the main factors (K^+^ treatments × time post-activation) was significant (*p* < 0.0001). Therefore, the effects of K^+^ were analyzed at each time post-activation, which were significant (*p* < 0.01; Figs 5D–5F and S1). In K^+^-free ASW, sperm velocity was significantly decreased at ≥15 min post-activation. In ASW containing 4-AP, a significant decrease in sperm velocity was observed in a concentration-dependent manner, which was significant at ≥10 mM at 1 min post-activation and 50 mM at 5 and 15 min post-activation. There were no significant differences in sperm velocity in ASW containing 50–500 μM glybenclamide within 60 min of the motility period.

These results show decreases in sperm motility and velocity in K^+^-free ASW and suggest contributions of a K^+^ channel in sperm motility regulation.

### Effect of Ca^2+^ on sperm kinematics

**Fig 6:**
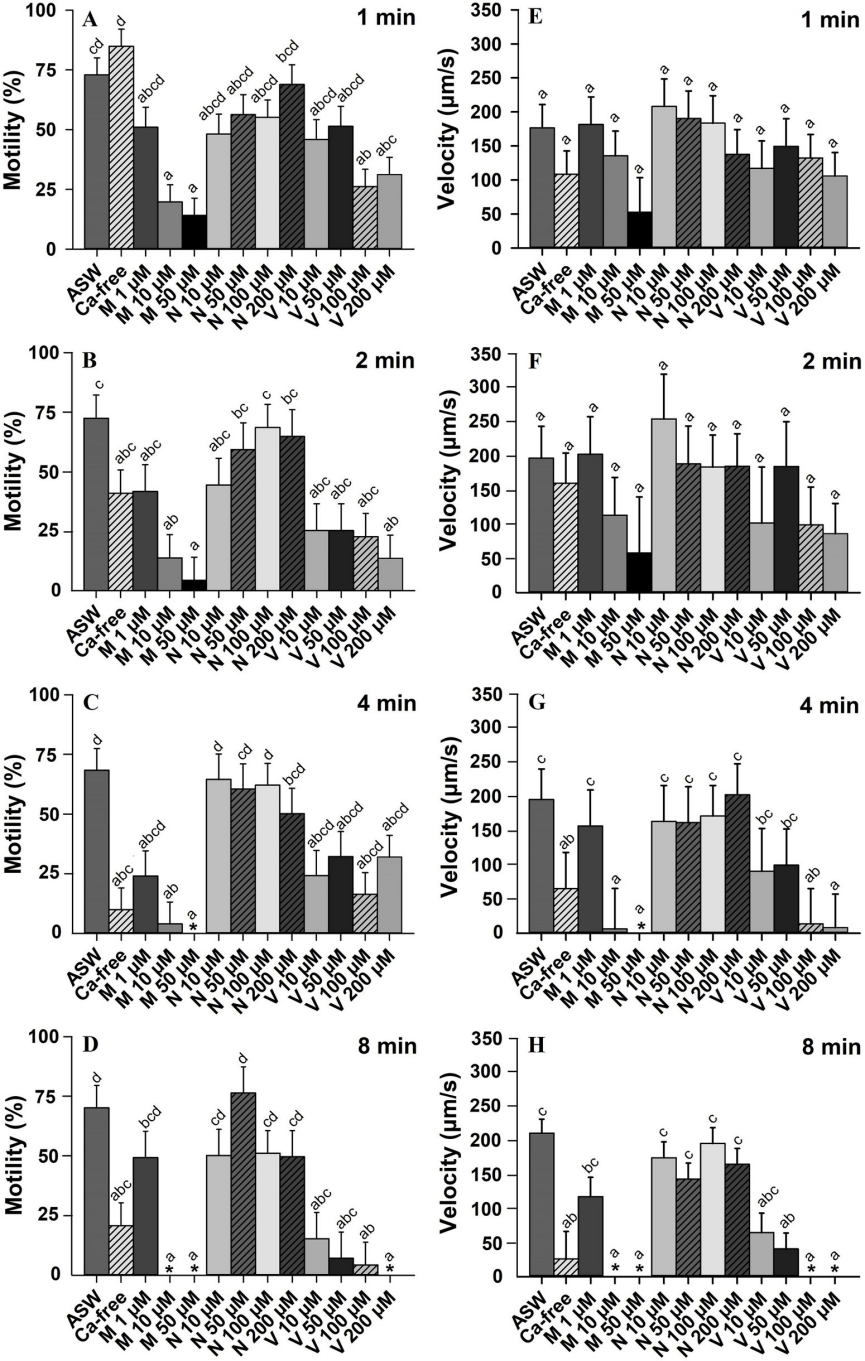
Effect of calcium (Ca^2+^) ions on sperm motility (%, A-D) and velocity (μm/s, E-H) in Eastern oyster, *Crassostrea virginica*. Sperm was activated in artificial seawater (ASW), Ca^2+^-free ASW and ASW containing Ca^2+^ channel blockers: mibefradil (M), nifedipine (N), or verapamil (V). Data were analyzed using a repeated measures ANOVA and shown as mean ± SE (n = 4). Treatments with different superscripts significantly differ (*p* < 0.05). Motility of 0% and velocity of 0 μm/s were indicated by asterisk.

To understand the contribution of Ca^2+^ ions in sperm motility, sperm were diluted in Ca^2+^-free ASW and in ASW containing Ca^2+^ channel blockers or EGTA. For both sperm motility and velocity, the interactions between the main factors (Ca^2+^ treatments × time post-activation) were significant (*p* < 0.001). Therefore, sperm motility and velocity were analyzed at each time post-activation, which were also significant (*p* < 0.0001) except for sperm velocity at 1 and 2 min post-activation (*p* > 0.05; Figs 6 and S2 and S3).

Sperm motility was initiated in Ca^2+^-free ASW, but significantly decreased at 4 to 20 min post-activation (Figs 6A–6D and S2). At 1 and 2 min post-activation, a decreasing trend in sperm velocity was observed after diluting sperm in Ca^2+^-free ASW, however the observed decrease was significant at 4 to 20 min post-activation (Figs 6E–6H and S3).

In the presence of Ca^2+^ channel blockers, sperm motility decreased in ASW containing ≥10 μM mibefradil at 1 and 2 min post-activation and ≥100 μM verapamil at 1 or 2 min post-activation (Fig 6A and 6B), while no significant changes in sperm velocity were observed (Fig 6E and 6F). At 4 min post-activation, sperm velocity was decreased in ASW containing ≥10 μM mibefradil (Fig 6G). By 8 min post-activation, sperm motility was not initiated at 10 and 50 μM mibefradil (Fig 6D). Verapamil decreased sperm motility at lower concentrations (10 or 50 μM) at 8 to 20 min post-activation (Figs 6D and S2). Also, at 8 to 20 min post-activation, sperm velocity was decreased in ASW containing ≥50 μM verapamil (Figs 6H and S3). Nifedipine (10 to 200 μM) was without effects on sperm motility and velocity (*p* > 0.05; Figs 6 and S2 and S3).

To chelate all traces of Ca^2+^ ions, sperm motility and velocity were recorded in ASW containing EGTA. For sperm motility, the interactions between the EGTA concentrations × time post-activation were significant (*p* < 0.01). Therefore, the effects of EGTA concentration on sperm motility were analyzed at each post-activation time, which were significant (*p* < 0.01; S4 Fig). At 1 and 2 min, sperm motility was initiated in ASW containing 0.5–3.5 mM EGTA (*p* > 0.05). Sperm motility was suppressed in ASW containing 3.5 mM mM EGTA at 6 and 8 min post-activation (S4 Fig). By 10 min post-activation a significant decrease in sperm motility was observed in ASW containing 3.0 and 3.5 mM EGTA (S5 Fig).

For sperm velocity, the interaction between EGTA concentrations × time post-activation was not significant (*p* > 0.05; S5 Fig), thus the effects of EGTA concentration and time post-activation were interpreted. Sperm velocity was affected by EGTA concentrations with significant decreases observed at ≥2.5 mM EGTA (*p* < 0.0001; S5 Fig). Sperm velocity was affected by time post-activation with highest values at 2 min compared to 6, 10, and 16 min post-activation (*p* < 0.001; S5 Fig).

Results show inhibition of sperm motility and a decrease in sperm velocity after activation of sperm in Ca^2+^-free ASW, ASW containing EGTA or ASW containing mibefradil and verapamil. These suggest that [Ca^2+^]_e_ is required for sperm motility regulation.

### Effect of Na^+^ on sperm kinematics

**Fig 7:**
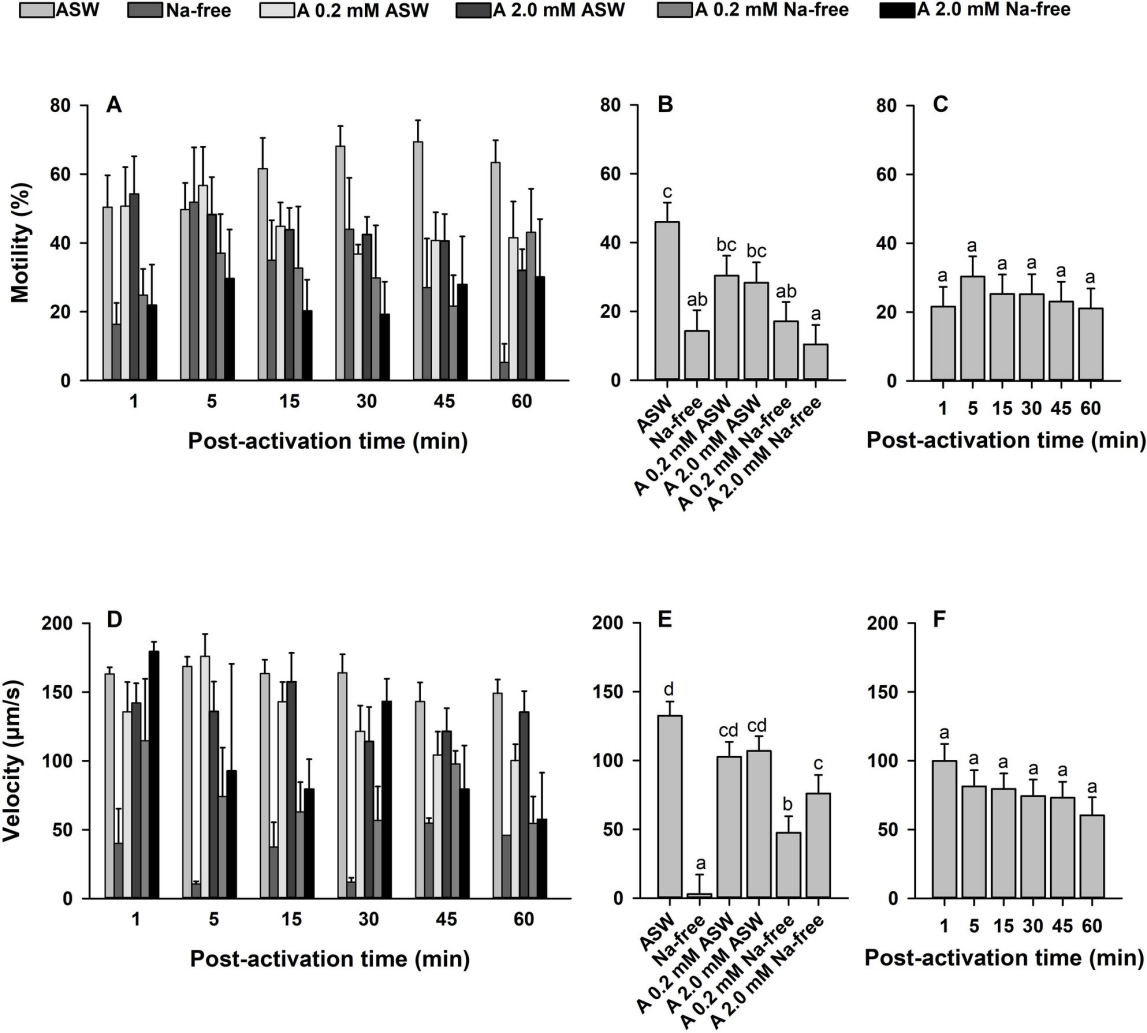
Effect of sodium (Na^+^) ions on sperm motility (%, A-C) and velocity (μm/s, D-F) in Eastern oyster, *Crassostrea virginica*. Sperm was activated in artificial seawater (ASW), Na^+^-free ASW and Na^+^-free ASW or ASW containing Na^+^ channel blockers: amiloride (A). Motility and velocity with each treatment (B, E) and time post-activation (C, F) is displayed. Data were analyzed using a repeated measures ANOVA and shown as mean ± SE (n = 5). Treatments with different superscripts significantly differ (*p* < 0.05).

To understand the contribution of Na^+^ ions the cells were assessed in Na^+^-free ASW with or without Na^+^ channel blockers. The interactions between the main factors (Na^+^ treatments × time post-activation) were not significant for sperm motility and velocity (*p* > 0.05; Fig 7A and 7D). Therefore, the effects of Na^+^ treatments and time post-activation were interpreted. There were effects of Na^+^ ions on sperm motility and velocity (*p* < 0.0001), which were decreased in Na^+^-free ASW containing ≥0.02 mM amiloride (Fig 7B and 7E). However, time post-activation did not affect sperm motility and velocity (*p* > 0.05; Fig 7C and 7F).

### Effect of Mg^2+^ on sperm kinematics

**Fig 8:**
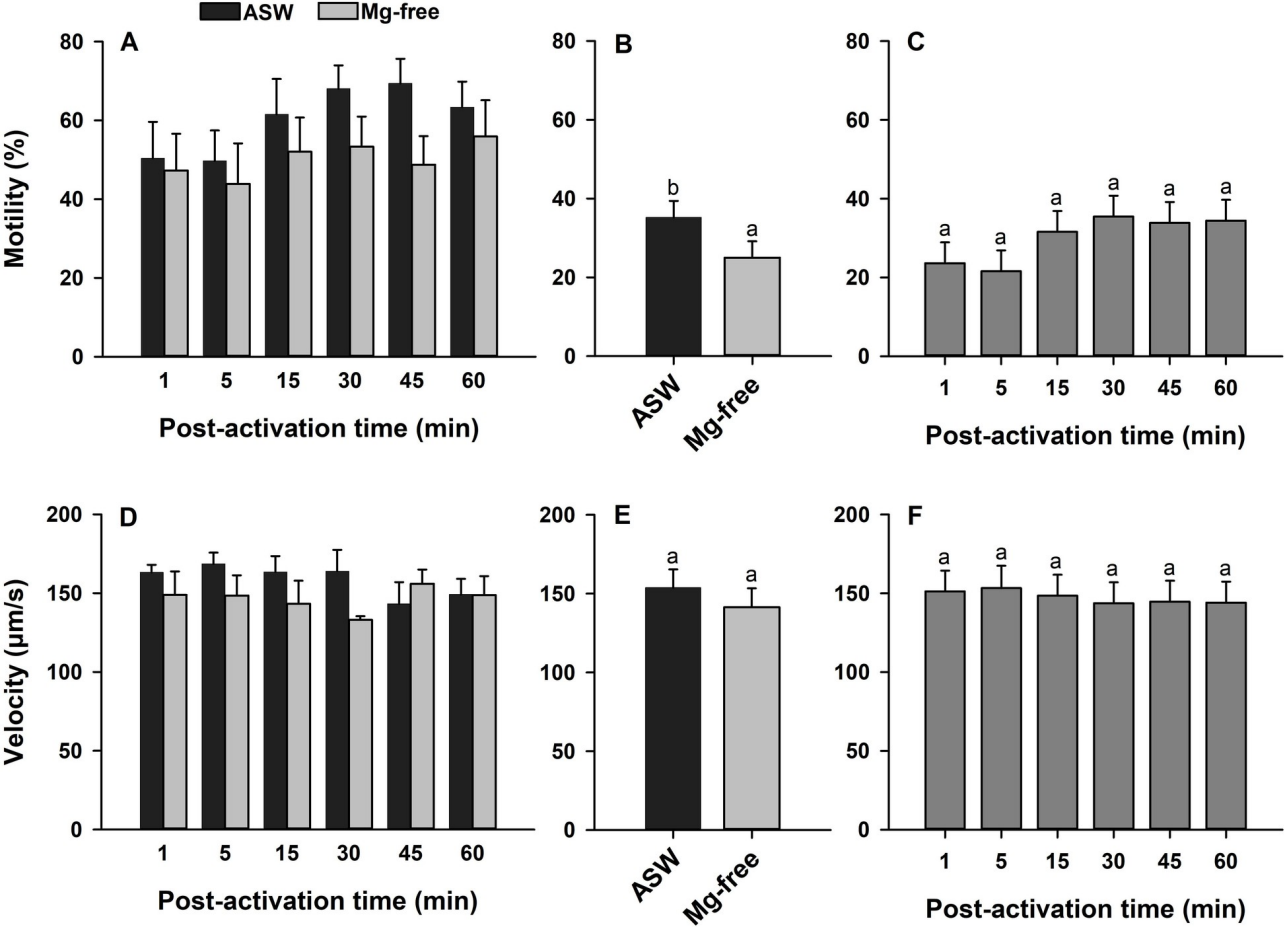
Effect of magnesium (Mg^2+^) ions on sperm motility (%, A-C) and velocity (μm/s, D-F) in Eastern oyster, *Crassostrea virginica*. Sperm was activated in artificial seawater (ASW) or Mg^2+^-free ASW. Motility and velocity with each treatment (B, E) and time post-activation (C, F) is displayed. Data were analyzed using a repeated measures ANOVA and shown as mean ± SE (n = 5). Treatments with different superscripts significantly differ (*p* < 0.05).

Finally, to understand the contribution of Mg^2+^ ions on sperm motility and velocity, the cells were assessed in Mg^2+^-free ASW. The interactions between the main factors (Mg^2+^ treatments and time post-activation) were not significant for sperm motility and velocity (*p* > 0.05; Fig 8A and 8D). Therefore, the effects of Mg^2+^ and time post-activation were interpreted. There were effects of Mg^2+^ on sperm motility, which were decreased in Mg^2+^-free ASW (*p* < 0.01; Fig 8B), while velocity did not differ (*p* > 0.05; Fig 8E). Time post-activation did not affect sperm motility and velocity (*p* > 0.05; Fig 8C and 8F).

## Discussion

Although the molecular structure of the motility apparatus is evolutionarily conserved in sperm of animals [66], the motility regulatory mechanisms still show diversity [1,2,4,6,9,67]. In this context, regulation of sperm motility is largely unknown in bivalves, and limited to species that typically spawn at higher salinities or in full-strength seawater [5]. In the present study, we investigated regulation of sperm motility activation in the Eastern oyster that spawns across a wide range of salinities (~10–30 PSU) under field and laboratory conditions [42–44]. Sperm motility was activated in seawater with salinity ranging from 4 to 32 PSU and was suppressed in seawater with salinity of >32 PSU. To study physiological effects of pH and ions, sperm motility and velocity were assessed in ASW with salinity of 20 PSU at pH 5.0–11.0, and in ionic-free ASW. Sperm motility was inhibited in ASW with pH ≤7.0, and in Ca^2+^, Na^+^, K^+^, and Mg^2+^-free ASW; associated with decreases in sperm velocity except for Mg^2+^-free ASW, suggesting that [Ca^2+^]_e_, [Na^+^]_e_, [K^+^]_e_, and [Mg^2+^]_e_ contribute to sperm motility regulation. Applications of Ca^2+^, Na^+^, and K^+^ channel blockers resulted in decreases of sperm motility and velocity, suggesting that ionic fluxes occur in sperm motility activation. Results show that regulation of sperm motility in the Eastern oyster share similarities with those of bivalves that spawn in normal seawater [28,33,36–40].

Both sperm motility and velocity were affected by the salinity of seawater (Figs 1 and 2). Osmolality of the testicular fluid (569.6 ± 34.6 mOsmol/kg) was within the range of osmolality of seawater in which sperm motility was activated in the Eastern oyster: 4 PSU (32.67 mOsmol/kg) and 32 PSU (850.00 mOsmol/kg). Paniagua-Chavez et al. [41] also reported similar osmolality for the testicular fluid of Eastern oyster (573 ± 45 mOsmol/kg) and observed that sperm could be activated in seawater with osmolality ranging from 203 to 833 mOsmol/kg. In the Pacific oyster, Dong et al. [33] and Boulais et al. [36] reported that sperm motility was activated in seawater with osmolality of 400–1400 mOsmol/kg and salinity of 14–50 PSU, respectively, while osmolality of testicular fluid was 1060 mOsmol/kg. These findings show that sperm motility is also activated in seawater with osmolality lower than that of testicular fluid. Therefore, osmolality of the testicular fluid is not a key factor to maintain sperm in the quiescent state in the reproductive organ of bivalves including Eastern oyster.

We observed the highest sperm motility and velocity in seawater with salinity of 16.83 and 17.91 PSU (Figs 1D and 2D), which possess osmolality of 338–447 mOsmol/kg (S2 Table). Compared to our observation, Paniagua-Chavez et al. [41] reported that sperm motility was higher in 833 mOsmol/kg ASW than 203, 403, and 601 mOsmol/kg ASW. Similar to Paniagua-Chavez et al. [41], it has been reported that sperm of Pacific oyster collected from ambient seawater shows the highest motility and velocity at 19.3 to 42.7 PSU [36] and 800–1100 mOsmol/kg [33]. Differences in highest sperm motility and velocity between this study and that of Dong et al. [33] and Boulais et al. [36] suggest that the osmolality range allowing the highest sperm kinematics broadened and shifted toward higher values with increasing ambient salinity as reported for euryhaline fishes [68–70].

We observed that the testicular fluid pH is 5.80 ± 0.04 in Eastern oyster. Sperm motility was suppressed in ASW pH ≤6.0, and sperm motility and velocity were highest in ASW with pH 7.5–10.0. Acidic pH of testicular fluid, inhibition of sperm motility in acidic ASW, and highest motility kinetics in alkaline ASW have been reported for Japanese pearl, Pacific, black-lip pearl and European flat oysters, manila clam, and great and Japanese scallops [28,36–40]. These results indicate that acidic pH of testicular fluid contributes to maintain sperm in the quiescent state in the reproductive organ of bivalves including Eastern oyster. Furthermore, higher percentages of motile sperm and sperm velocity were observed in ASW with pH 8.66–9.09 (Figs 3D and 4D), which is consistent with previous studies on bivalves [28,36–40]. This could be due to the fact that environmental alkaline pH rises the [pH]_i_ in which dynein ATPase activity is stimulated [50,71,72]. It is worth noting that Prytherch [46] reported decreases in Eastern oyster reproductive success when environmental pH was decreased as the sperm and oocytes lose their receptivity within 2 to 4 h outside of pH ranging from 6.0 to 10.0 [73].

Previous reports on the Pacific oyster, Japanese scallop, and Manila clam indicated that sperm motility activation in bivalves requires ionic fluxes [28]. Similarly, we also observed that Eastern oyster sperm motility (n = 3 males) was not activated in 0.5 or 1.0 M sucrose, 10 mM Tris, pH 8.0 solutions supplemented with 0.4% Pluronic (data not shown). Therefore, further experiments were performed to study regulatory roles of ions in sperm motility regulation.

Eastern oyster sperm motility was activated in K^+^-free ASW, however the percentage of motility and sperm velocity were decreased at 15 min post-activation (Fig 5). Similarly, Boulais et al. [36] reported no change in sperm motility and velocity in the Pacific oyster when sperm were activated in K^+^-free ASW within 5 min post-activation. Alavi et al. [28] reported that both sperm motility and velocity in the Japanese scallop were decreased in K^+^-free ASW at 1 min post-activation. These results suggest that K^+^ ions in seawater are important to activate sperm motility or create optimal conditions for maintenance of sperm motility in bivalves, similar to that of sea urchin [74]. Similar to Japanese scallop sperm [28], we observed that motility and velocity of Eastern oyster sperm were decreased in ASW containing 4-AP, which is a voltage-dependent K^+^ channel blocker [75]. This study also showed inhibition of sperm motility initiation in ASW containing glybenclamide, an ATP-sensitive K^+^ channel, which depolarizes the membrane potential [76]. These results may suggest an involvement of both voltage-dependent and ATP-sensitive K^+^ channels in sperm motility activation in bivalves. It has been reported that increases in [K^+^]_e_ concentrations result in inhibition of sperm motility and decreases in sperm velocity [28] associated with membrane depolarization (Alavi et al., unpublished data). Therefore, K^+^ efflux could be occurring upon sperm motility activation in bivalves including Eastern oyster; as has been demonstrated for mammals, fish, ascidian, and sea urchin [47,77–81].

We also observed that sperm velocity was decreased in K^+^-free ASW or in ASW containing 4-AP (Fig 5), which was consistent with a previous study on Japanese scallop sperm [28]. Alavi et al. [28] examined sperm motility initiation in the presence of nigericin which is an intracellular K^+^ ([K^+^]_i_) /extracellular H^+^ ([H^+^]_e_) exchanger. Under this condition, [pH]_i_ is equivalent to [pH]_e_ if [K^+^]_e_ is equivalent to [K^+^]_i_ [82], and H^+^ efflux occurs in sperm following activation in a solution with [pH]_e_ higher than [pH]_i_ [83]. Results showed that sperm motility was only initiated in ASW pH 8.3 containing nigericin [28]. Therefore, higher sperm motility and velocity in alkaline ASW, and lower sperm motility and velocity in K^+^-free ASW or ASW containing 4-AP suggests that K^+^ ions might be involved in regulation of [pH]_i_.

Sperm motility and velocity in the Eastern oyster was initiated in Ca^2+^-free ASW or in ASW containing EGTA, however they were rapidly decreased compared to ASW (Figs 6 and S2 and S4). This confirms previous statements that sperm longevity is Ca^2+^-dependent in bivalves [5]. At 6 min post-activation, sperm motility in Eastern oyster, Pacific oyster, and Japanese scallop were suppressed in ASW containing 3.5, 5.0, and 10 mM EGTA, respectively [28, this study], suggesting that it might be a species-specific requirement to [Ca^2+^]_e_ ions. It has been shown that the Ca^2+^ required for sperm motility initiation is higher in euryhaline fish reared at higher salinity [68,70,84].

Inhibition or suppression of sperm motility in the presence of EGTA, mibefradil or verapamil (Figs 6 and S2 and S4) suggests that sperm of the Eastern oyster requires Ca^2+^ influx for sperm motility; as reported in bivalves, sea urchin, ascidian, and fish [51,54,74,85–87]. In bivalves, activation of sperm motility was shown to be associated with [Ca^2+^]_i_ rise, which was not observed in the presence of EGTA or a Ca^2+^ channel blocker including mibefradil [28]. Similar to Japanese scallop [28], 50 μM mibefradil was capable of suppressing sperm motility within 4–6 min post activation. Verapamil at >100 μM suppressed sperm motility activation in the Eastern oyster at 6 min post-activation, however Alavi et al. [28] reported inhibition of sperm motility in the Japanese scallop after activation in ASW containing 200 μM verapamil at 120 min post-activation. Moreover, we observed that nifedipine (10–200 μM) did not impact sperm motility in the Eastern oyster, but it caused a decrease in sperm motility of Japanese scallop at 15 min and suppressed it at 120 min post-activation [28]. These differences may suggest the presence of species-specific types of Ca^2+^ channels in sperm of bivalves however, the molecular identity and characterization of Ca^2+^ channels are completely unknown in bivalves.

Sperm velocity in the Eastern oyster was also decreased in Ca^2+^-free ASW, ASW containing EGTA, mibefradil, and verapamil (Figs 6 and S3 and S5), similarly to other bivalve species [28], suggesting that Ca^2+^ ions are involved in flagellar beating force. It is worth to speculate that Alavi et al [28] also reported full suppression of sperm motility in bivalves after activation in ASW containing 100–200 μM W-7, an inhibitor for calmodulin protein phosphodiesterase.

A decrease in sperm motility after activation in Na^+^-free ASW (Fig 7) is consistent with previous observations on the Manila clam, Japanese scallop, and Pacific oyster [28,36,40], and suggests that [Na^+^]_e_ is essential for sperm motility. Inhibition of sperm motility and velocity in the presence of amiloride suggests that a Na^+^ channel is involved in sperm motility activation. Previous studies have suggested that [Na^+^]_e_ ions may contribute to regulate [pH]_i_ via a Na^+^/K^+^-ATPase or a Na^+^/H^+^ exchanger, causing an alkalization that is required for sperm motility activation [36,56,88]. It might be possible that Na^+^ ions regulate [Ca^2+^]_i_ ions via Na^+^/Ca^2+^ exchanger [28,86,87]. It is worth noting that amiloride did not affect sperm motility or velocity of the Eastern oyster, when it was added into ASW. This might be due to low [Ca^2+^]_e_ requirements for sperm motility activation. In this regard, Faure et al. [40] reported that full depletion of [Na^+^]_e_ totally suppresses sperm motility activation in scallop.

Out of all the ion free ASW solutions, Mg^2+^-free ASW showed a smaller effect on sperm kinematics (Fig 8) as has reported on the Pacific oyster sperm, suggesting that Mg^2+^ possesses a lower contribution to activation of sperm motility [36].

## Conclusions

The present study reveals insights into the cellular processes behind sperm motility activation in bivalves and provides vital information, which can be used by hatcheries to improve artificial fertilization. Sperm motility in Eastern oyster was activated in a wide range of salinities (4–32 PSU) and pH (6.5–10.5). Our results show that when sperm spawned into seawater, K^+^ ions flux occur through K^+^ channels including an ATP-sensitive K^+^ channel or a voltage-gated K^+^ channel. As above, based on membrane depolarization in the presence of high concentrations of [K^+^]_e_ [Alavi et al., unpublished data] K^+^ ions flux is outward. Sperm motility activation in alkaline seawater requires [Ca^2+^]_e_ and [Na^+^]_e_ ions as sperm motility was inhibited or suppressed in Ca^2+^- or Na^+^-free ASW. Influx of Ca^2+^ and Na^+^ ions are mediated by Ca^2+^ and Na^+^ channels, respectively. It has been previously suggested that Na^+^ and Ca^2+^ are involved in regulation of [pH]_i_ and activation of axonemal proteins, respectively [28,36]. Taken together, this study shows that ionic regulations of sperm motility activation are similar between the Eastern oyster spawning in seawater at lower salinities compared to other species of bivalves that typically spawn at higher salinities. However, differences observed in inhibitory effects of ion channel blockers may suggest specificity to extracellular ion requirements. This might be due to the impact of environmental salinity or due to species-specificity of ion channels. However, molecular identification and physiological characterization of specific ion channels are extensively unknown in bivalves and remain to be elucidated in further studies.

Ultimately, this study can be used to improve bivalve aquaculture by optimizing activation or immobilizing medium. For instance, to preserve sperm for short or long-term storage (cryopreservation), it can be maintained in the quiescent state in ASW close to that of testicular fluid. This study also has implications that surpass the scope of assisted reproduction into a more comprehensive understanding of the natural ecology of the Eastern oyster. For instance, our results provide ecologists with valuable information to better predict how changes in the ocean will impact oyster spawning dynamics.

## Supporting information

S1 Fig Effect of potassium (K+) ions on sperm velocity (μm/s) in Eastern oyster, Crassostrea virginica at 30 (A), 45 (B) and 60 (C) min post-activation. Sperm was activated in K+-free artificial seawater (ASW) and ASW containing a voltage-gated (4-aminopyridine, 4-AP) or an ATP-sensitive (glybenclamide, G) K+ channel blocker. Data were analyzed using a repeated measures ANOVA and shown as mean ± SE (n = 5). Treatments with different superscripts significantly differ (P < 0.05).
(TIF)

S2 Fig Effect of calcium (Ca2+) ions on sperm motility (%) in Eastern oyster, Crassostrea virginica at 6 (A), 10 (B), 12 (C), 14 (D), 16 (E), 18 (F), and 20 (G) min post-activation. Sperm was activated in artificial seawater (ASW), Ca2+-free ASW and ASW containing Ca2+ channel blockers: mibefradil (M), nifedipine (N), or verapamil (V). Data were analyzed using a repeated measures ANOVA and shown as mean ± SE (n = 4). Treatments with different superscripts significantly differ (P < 0.05). A motility of 0% was indicated by asterisk.
(TIF)

S3 Fig Effect of calcium (Ca2+) ions on sperm velocity (μm/s) in Eastern oyster, Crassostrea virginica at 6 (A), 10 (B), 12 (C), 14 (D), 16 (E), 18 (F), and 20 (G) min post-activation. Sperm was activated in artificial seawater (ASW), Ca2+-free ASW, and ASW containing Ca2+ channel blockers: mibefradil (M), nifedipine (N) or verapamil (V). Data were analyzed using a repeated measures ANOVA and shown as mean ± SE (n = 4). Treatments with different superscripts significantly differ (P < 0.05). A velocity of 0 μm/s was indicated by asterisk.
(TIF)

S4 Fig Sperm motility (%, A-K) in Eastern oyster, Crassostrea virginica after activation in artificial seawater containing EGTA. Data were analyzed using a repeated measures ANOVA and shown as mean ± SE (n = 4). Treatments with different superscripts significantly differ (P < 0.05). Motility of 0% was indicated by asterisk.
(TIF)

S5 Fig Sperm velocity (μm/s, A) in Eastern oyster, Crassostrea virginica after activation in artificial seawater containing EGTA. Average velocity at each EGTA concentration (B) and time post-activation (C) is displayed. Data were analyzed using a repeated measures ANOVA and shown as mean ± SE (n = 4). Treatments with different superscripts significantly differ (P < 0.05). Velocity of 0 μm/s was indicated by asterisk.
(TIF)

S1 Table Length, width, height, and weight of Eastern oyster, *Crassostrea virginica* used in the present study as well as sperm density. (DOCX)

S2 Table Salinities and corresponding osmolality for activation media used to study the effects of salinity on sperm motility kinematics in the Eastern oyster, *Crassostrea virginica*. (DOCX)

S3 Table Sperm activity parameters of the Eastern oyster, *Crassostrea virginica*, from the pH and salinity experiments modeled by second-order polynomial regressions. For each time, the equation of the line, R^2^ value, and absolute maximum or peak (vertex) are shown in columns for both motility and velocity. Significant values are denoted by ***P < 0.*0001* and **P < 0.*01*.
(DOCX)

S1 Data OysterSperm Data 25 Oct 2020 FINALv2. (XLSX)
