# Supplementary figures and images for: Regulation of sperm motility in Eastern oyster (Crassostrea virginica) spawning naturally in seawater with low salinity
Source: PLoS One. 2021 Mar 18;16(3):e0243569. doi: 10.1371/journal.pone.0243569 (PMC7971463; doi:10.1371/journal.pone.0243569)

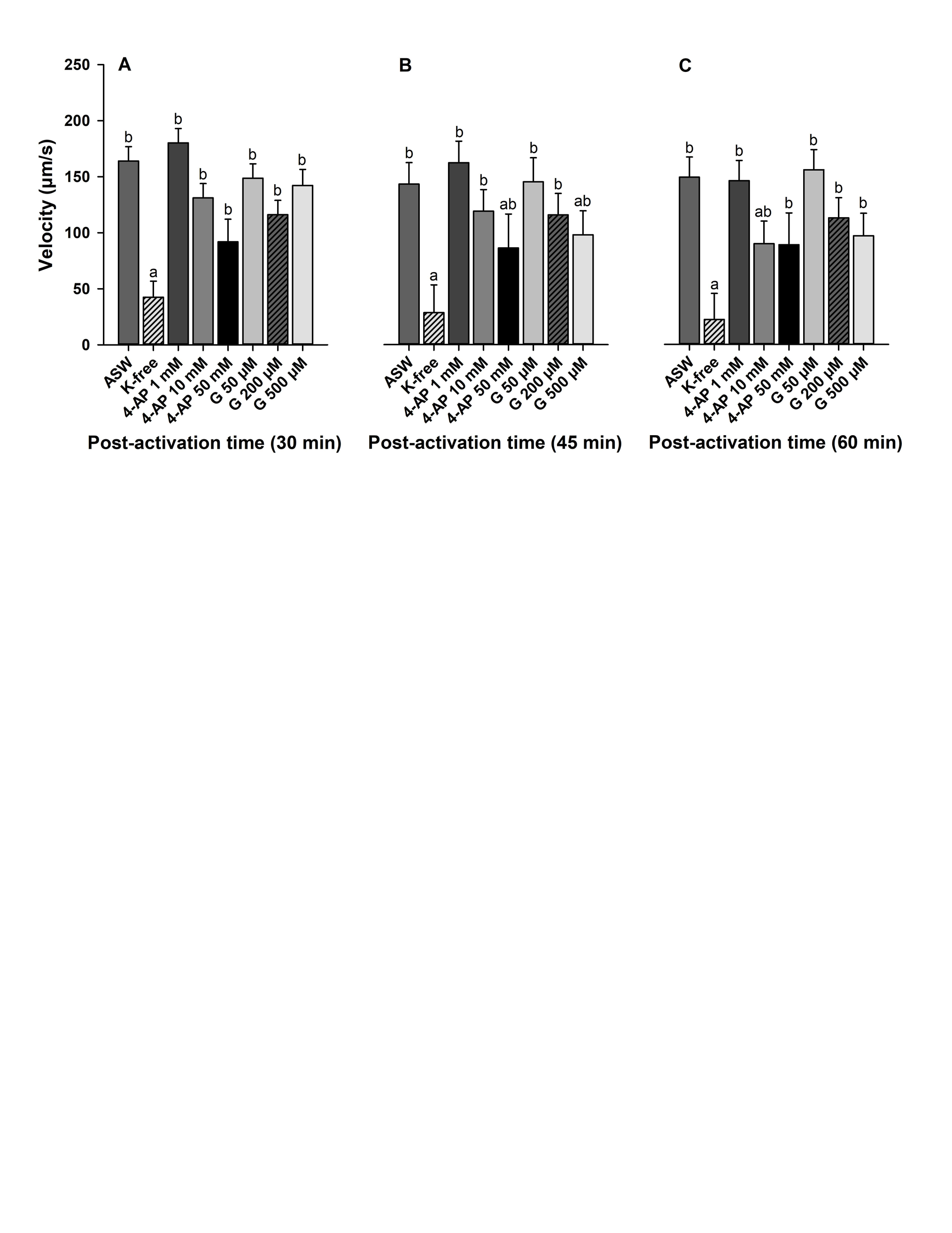

Supplement: S1 Fig — Effect of potassium (K+) ions on sperm velocity (μm/s) in Eastern oyster, Crassostrea virginica at 30 (A), 45 (B) and 60 (C) min post-activation. Sperm was activated in K+-free artificial seawater (ASW) and ASW containing a voltage-gated (4-aminopyridine, 4-AP) or an ATP-sensitive (glybenclamide, G) K+ channel blocker. Data were analyzed using a repeated measures ANOVA and shown as mean ± SE (n = 5). Treatments with different superscripts significantly differ (P < 0.05). (TIF) [file pone.0243569.s001.tif]

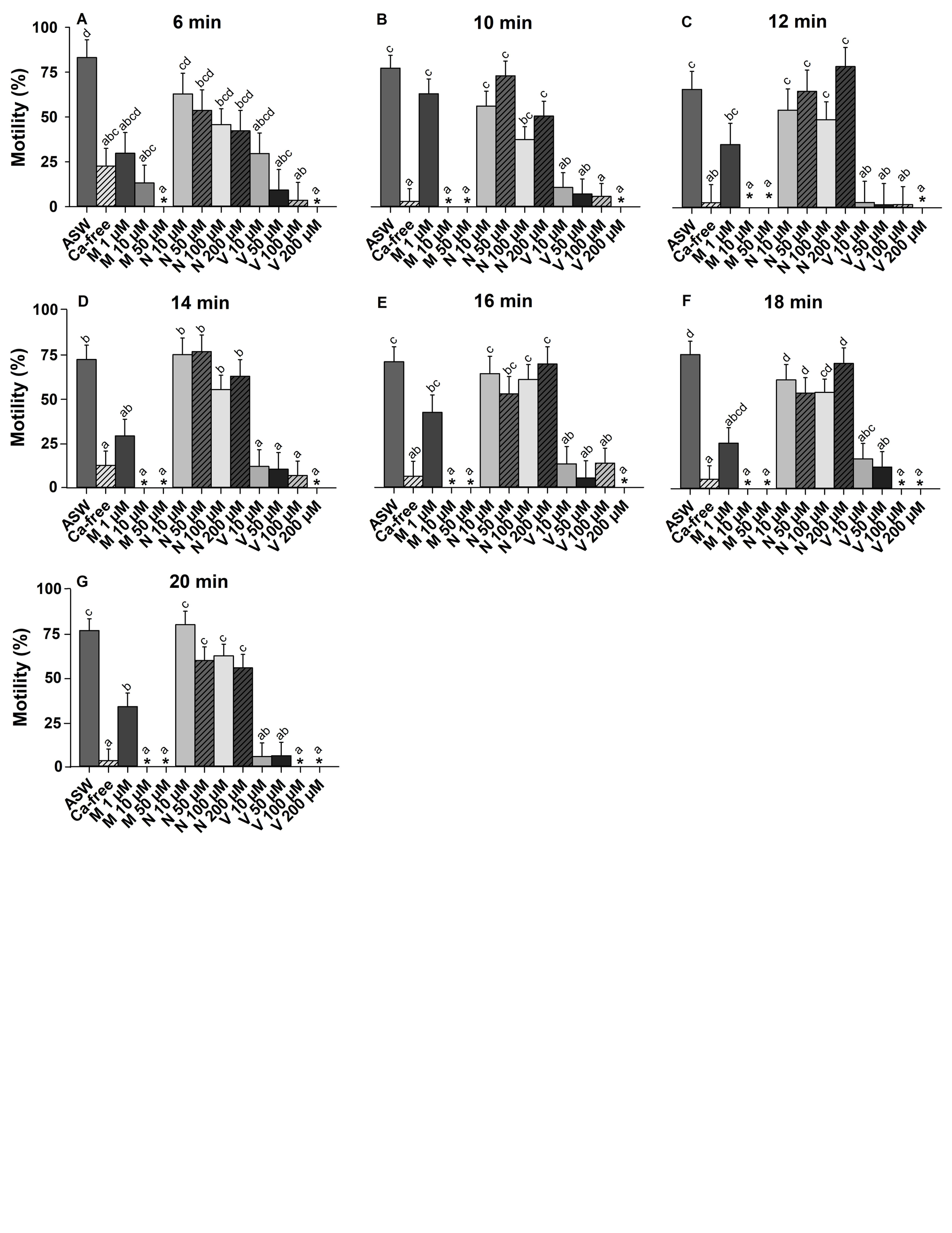

Supplement: S2 Fig — Effect of calcium (Ca2+) ions on sperm motility (%) in Eastern oyster, Crassostrea virginica at 6 (A), 10 (B), 12 (C), 14 (D), 16 (E), 18 (F), and 20 (G) min post-activation. Sperm was activated in artificial seawater (ASW), Ca2+-free ASW and ASW containing Ca2+ channel blockers: mibefradil (M), nifedipine (N), or verapamil (V). Data were analyzed using a repeated measures ANOVA and shown as mean ± SE (n = 4). Treatments with different superscripts significantly differ (P < 0.05). A motility of 0% was indicated by asterisk. (TIF) [file pone.0243569.s002.tif]

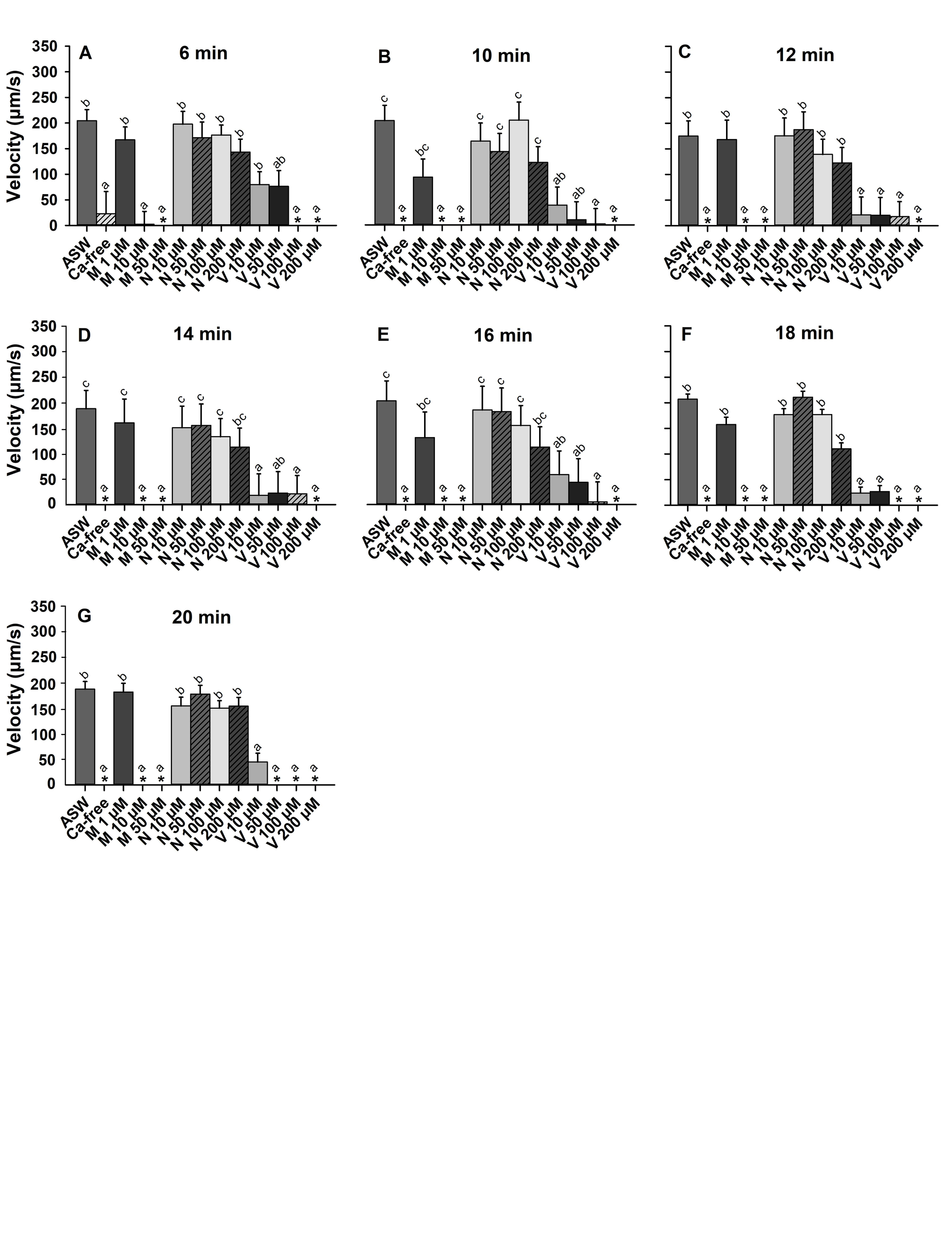

Supplement: S3 Fig — Effect of calcium (Ca2+) ions on sperm velocity (μm/s) in Eastern oyster, Crassostrea virginica at 6 (A), 10 (B), 12 (C), 14 (D), 16 (E), 18 (F), and 20 (G) min post-activation. Sperm was activated in artificial seawater (ASW), Ca2+-free ASW, and ASW containing Ca2+ channel blockers: mibefradil (M), nifedipine (N) or verapamil (V). Data were analyzed using a repeated measures ANOVA and shown as mean ± SE (n = 4). Treatments with different superscripts significantly differ (P < 0.05). A velocity of 0 μm/s was indicated by asterisk. (TIF) [file pone.0243569.s003.tif]

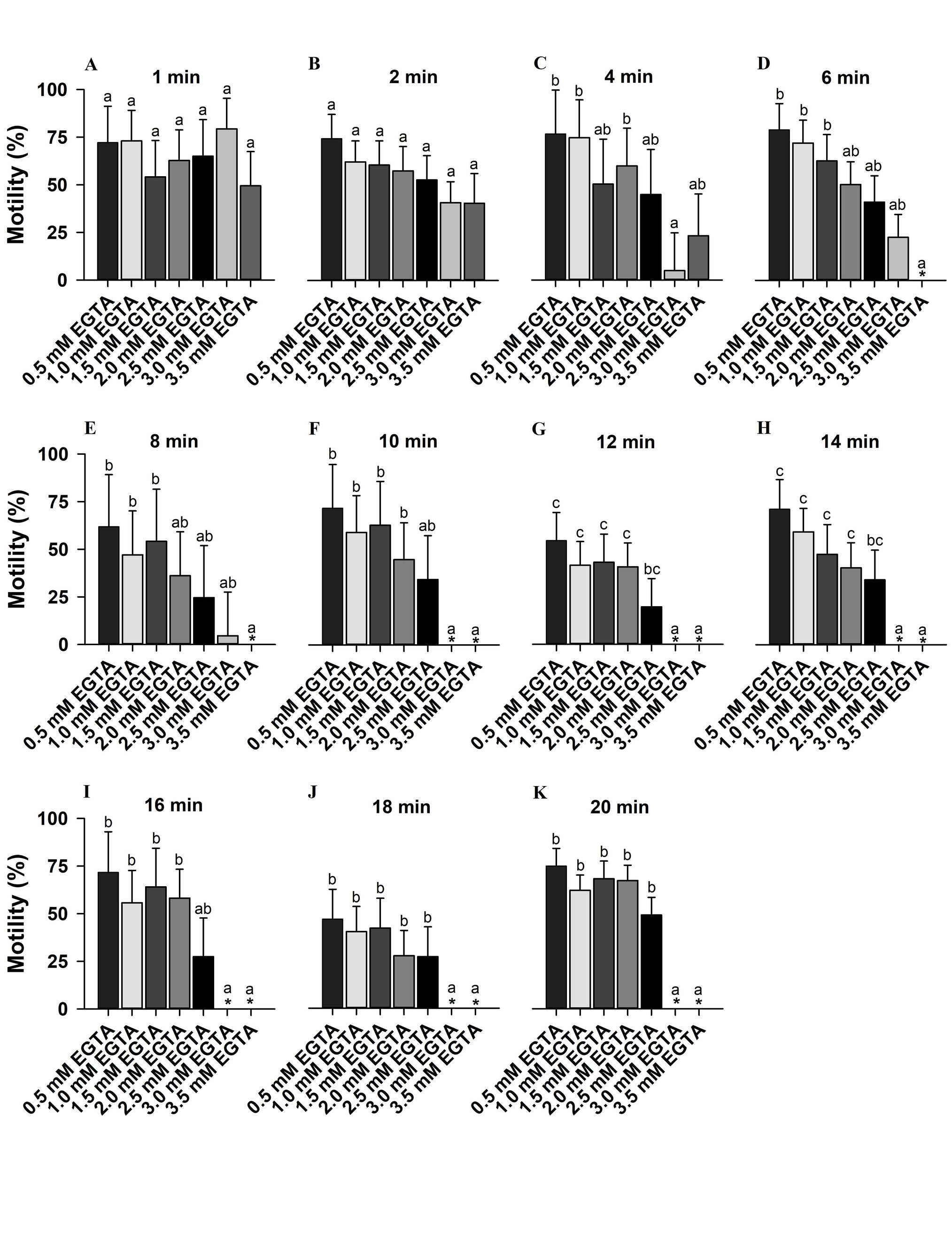

Supplement: S4 Fig — Sperm motility (%, A-K) in Eastern oyster, Crassostrea virginica after activation in artificial seawater containing EGTA. Data were analyzed using a repeated measures ANOVA and shown as mean ± SE (n = 4). Treatments with different superscripts significantly differ (P < 0.05). Motility of 0% was indicated by asterisk. (TIF) [file pone.0243569.s004.tif]

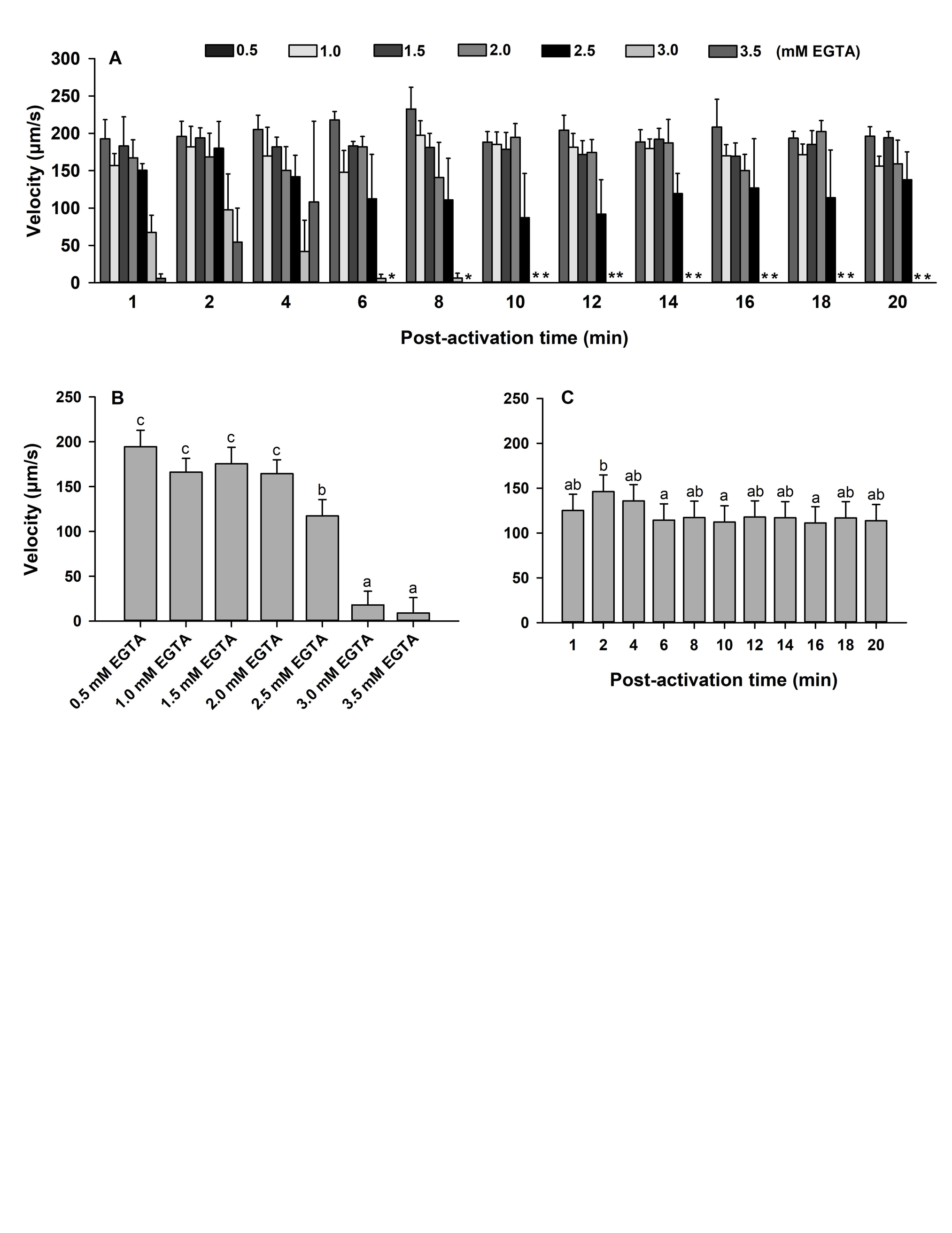

Supplement: S5 Fig — Sperm velocity (μm/s, A) in Eastern oyster, Crassostrea virginica after activation in artificial seawater containing EGTA. Average velocity at each EGTA concentration (B) and time post-activation (C) is displayed. Data were analyzed using a repeated measures ANOVA and shown as mean ± SE (n = 4). Treatments with different superscripts significantly differ (P < 0.05). Velocity of 0 μm/s was indicated by asterisk. (TIF) [file pone.0243569.s005.tif]
