# Supplementary material for: Regulation of sperm motility in Eastern oyster (Crassostrea virginica) spawning naturally in seawater with low salinity
Source: PLoS One. 2021 Mar 18;16(3):e0243569. doi: 10.1371/journal.pone.0243569 (PMC7971463; doi:10.1371/journal.pone.0243569)
Supplement: S1 Table — (DOCX) [file pone.0243569.s006.docx]

**Supplemental Table S1.** Length, width, height, and weight of Eastern oyster, *Crassostrea virginica* used in the present study as well as sperm density.

|  | **Oyster** | **Shell length (mm)** | **Shell width (mm)** | **Shell height (mm)** | **Total weight (g)** | **Sperm density (cells/mL)** |
| --- | --- | --- | --- | --- | --- | --- |
| Effect of pH | 1 | 50.10 | 23.20 | 69.40 | 48.16 | 9.40E+08 |
|  | 2 | 52.60 | 26.70 | 65.50 | 49.15 | 6.23E+08 |
|  | 3 | 45.00 | 26.20 | 67.50 | 48.19 | 9.53E+08 |
|  | 4 | 49.60 | 26.10 | 66.80 | 52.02 | 1.29E+09 |
| Effect of salinity | 1 | 55.76 | 36.10 | 96.65 | 128.03 | 3.51E+09 |
|  | 2 | 58.51 | 32.40 | 106.66 | 140.20 | 6.03E+09 |
|  | 3 | 59.20 | 34.90 | 89.48 | 115.71 | 2.76E+09 |
|  | 4 | 57.07 | 27.14 | 108.91 | 129.10 | 4.26E+09 |
|  | 5 | 57.97 | 37.08 | 84.30 | 90.18 | 2.34E+09 |
| Effect of Ca^2+^ | 1 | 66.09 | 36.45 | 92.73 | 135.05 | 5.12E+10 |
|  | 2 | 60.85 | 41.64 | 88.06 | 146.87 | 5.53E+10 |
|  | 3 | 56.41 | 33.90 | 94.76 | 103.90 | 2.81E+10 |
|  | 4 | 60.19 | 38.25 | 95.46 | 146.53 | 3.92E+10 |
| Effect of K^+^, Na^+^, Mg^2+^ | 1 | 67.20 | 34.50 | 95.40 | 116.85 | 1.60E+09 |
|  | 2 | 65.20 | 36.70 | 113.60 | 143.42 | 1.89E+10 |
|  | 3 | 67.80 | 45.50 | 100.40 | 145.71 | 4.98E+09 |
|  | 4 | 57.20 | 39.30 | 97.20 | 127.26 | 1.72E+10 |
|  | 5 | 63.10 | 42.30 | 92.70 | 146.14 | 4.46E+09 |

Sperm density was counted twice and averaged for each male.
