# Supplementary material for: Regulation of sperm motility in Eastern oyster (Crassostrea virginica) spawning naturally in seawater with low salinity
Source: PLoS One. 2021 Mar 18;16(3):e0243569. doi: 10.1371/journal.pone.0243569 (PMC7971463; doi:10.1371/journal.pone.0243569)
Supplement: S2 Table — (DOCX) [file pone.0243569.s007.docx]

**Supplemental Table S2.** Salinities and corresponding osmolality for activation media used to study the effects of salinity on sperm motility kinematics in the Eastern oyster, *Crassostrea virginica*.

|  |  |
| --- | --- |
| **Salinity**  **(PSU)** | **Osmolality (mOsmol/kg)** |
| 4 | 32.67 |
| 8 | 85.33 |
| 12 | 241.33 |
| 16 | 338.33 |
| 20 | 447.33 |
| 24 | 570.00 |
| 28 | 719.00 |
| 32 | 850.00 |
