# Supplementary material for: Regulation of sperm motility in Eastern oyster (Crassostrea virginica) spawning naturally in seawater with low salinity
Source: PLoS One. 2021 Mar 18;16(3):e0243569. doi: 10.1371/journal.pone.0243569 (PMC7971463; doi:10.1371/journal.pone.0243569)
Supplement: S3 Table — For each time, the equation of the line, R2 value, and absolute maximum or peak (vertex) are shown in columns for both motility and velocity. Significant values are denoted by ***P < 0.0001 and **P < 0.01. (DOCX) [file pone.0243569.s008.docx]

**Supplemental Table S3.** Sperm activity parameters of the Eastern oyster, *Crassostrea virginica*, from the pH and salinity experiments modeled by second-order polynomial regressions. For each time, the equation of the line, R^2^ value, and absolute maximum or peak (vertex) are shown in columns for both motility and velocity. Significant values are denoted by ****P < 0.0001* and ***P < 0.01*.
